# Validation of existing risk scores for mortality prediction after a heart transplant in a Chinese population

**DOI:** 10.1093/icvts/ivab380

**Published:** 2022-01-08

**Authors:** Shanshan Zheng, Hanwei Tang, Zhe Zheng, Yunhu Song, Jie Huang, Zhongkai Liao, Sheng Liu

**Affiliations:** 1 Department of Cardiac Surgery, Fuwai Hospital, National Center for Cardiovascular Diseases, Chinese Academy of Medical Sciences (CAMS) and Peking Union Medical College (PUMC), Beijing, China; 2 Department of Heart Failure and Heart Transplant, Fuwai Hospital, National Center for Cardiovascular Diseases, Chinese Academy of Medical Sciences (CAMS) and Peking Union Medical College (PUMC), Beijing, China

**Keywords:** Heart transplant, UNOS, IMPACT, RSS, Risk scores, Post-transplant mortality

## Abstract

**OBJECTIVES:**

The objectives of this study were to validate 3 existing heart transplant risk scores with a single-centre cohort in China and evaluate the efficacy of the 3 systems in predicting mortality.

**METHODS:**

We retrospectively studied 428 patients from a single centre who underwent heart transplants from January 2015 to December 2019. All patients were scored using the Index for Mortality Prediction After Cardiac Transplantation (IMPACT) and the United Network for Organ Sharing (UNOS) and risk stratification scores (RSSs). We assessed the efficacy of the risk scores by comparing the observed and the predicted 1-year mortality. Binary logistic regression was used to evaluate the predictive accuracy of the 3 risk scores. Model discrimination was assessed by measuring the area under the receiver operating curves. Kaplan–Meier survival analyses were performed after the patients were divided into different risk groups.

**RESULTS:**

Based on our cohort, the observed mortality was 6.54%, whereas the predicted mortality of the IMPACT and UNOS scores and the RSSs was 10.59%, 10.74% and 12.89%, respectively. Logistic regression analysis showed that the IMPACT [odds ratio (OR), 1.25; 95% confidence interval (CI), 1.15–1.36; *P* < 0.001], UNOS (OR, 1.68; 95% CI, 1.37–2.07; *P* < 0.001) and risk stratification (OR, 1.61; 95% CI, 1.30–2.00; *P* < 0.001) scores were predictive of 1-year mortality. The discriminative power was numerically higher for the IMPACT score [area under the curve (AUC) of 0.691)] than for the UNOS score (AUC 0.685) and the RSS (AUC 0.648).

**CONCLUSIONS:**

We validated the IMPACT and UNOS scores and the RSSs as predictors of 1-year mortality after a heart transplant, but all 3 risk scores had unsatisfactory discriminative powers that overestimated the observed mortality for the Chinese cohort.

**Figure:**
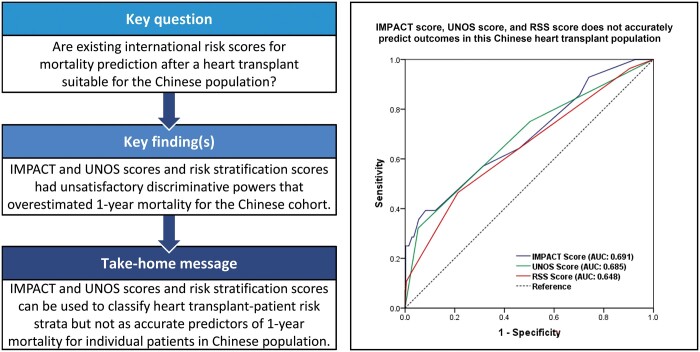

## INTRODUCTION

In the past decade, the number of patients listed for cardiac transplants has increased considerably, but the number of available organs is inadequate [1–3]. Because of the limited donor supply, optimizing recipient selection and recipient/donor matching are more important than ever [4–8]. In the global context, the identification of pretransplant risk factors has been an important part of the prognostic research for heart transplants (HTx). Risk score models have been proposed to predict the prognosis of patients undergoing an HTx [9–15]. Risk score tools can be used for clinical evaluation, decision-making, care and research for the HTx population, which may affect organ allocation [16]. Therefore, it is necessary to have an effective scoring tool for assessing postoperative risk in patients undergoing an HTx.

Various risk evaluation systems have been developed to predict heart-transplant mortality. The Index for Mortality Prediction After Cardiac Transplantation (IMPACT) (through 12 recipient-specific variables) is used to predict the likelihood of 1-year mortality for patients undergoing an HTx [12]. The HTx risk scoring model is based on United Network for Organ Sharing (UNOS) data (called UNOS score in this article), which is a scoring system based on recipient and donor risk factors used to predict post-transplant 1-year survival [17]. The preoperative risk stratification score (RSS) is a risk score based on recipient, donor and recipient-donor pairing factors for predicting graft failure at 1 year [15]. These 3 risk scores were all established by using the UNOS data and were well received in varying degrees for clinical application.

Currently, there is no preoperative risk score for a HTx based on a Chinese population cohort. Determining whether the existing risk scoring models are applicable to the Chinese population has not been verified. Therefore, the purpose of this study was to use a single-centre HTx cohort from Fuwai Hospital to validate the efficacy of the 3 risk scores, compare their performances and identify their applicability in Chinese HTx populations.

## METHODS

### Ethics statement

The study was approved by the ethical committee of Fuwai Hospital (Approval No. 2017-887). Patient consent was obtained from all who participated in the preoperative survey.

### Data source

Information for all patients was retrospectively collected from electronic medical records. Data were collected from January 2015 through December 2020. Information about the evaluation of the 3 risk scoring models included patient demographics, preoperative risk factors, postoperative course, morbidity and mortality.

### Study population

In this single-centre retrospective study, we included patients undergoing a single-organ HTx between January 2015 and December 2019. We excluded patients younger than 18 years of age (*n* = 21) and patients missing information on the risk score evaluation forms (*n* = 2). Patients were followed up from the date of the transplant until death, retransplant or the date of the last known follow-up. Follow-up data were provided through 31 December 2020. Our median follow-up time was 3.6 (1.0–6.2) years, and all patients finished at least 1 year of follow-up.

### Data analysis

The IMPACT and UNOS scores and the RSSs were calculated for each HTx recipient. The variables of the IMPACT and UNOS scores and the RSSs are shown in Table 1. We first compared the differences in baseline characteristics between Chinese and UNOS recipients according to the limited, original data provided by these 3 risk scores. Due to the lack of donor-characteristic data from the original article on the UNOS population, we compared the donor characteristics with those from the International Society of Heart and Lung Transplantation (ISHLT) population. Then, we ascertained each patient’s predicted 1-year mortality by the 3 risk scoring systems and measured the operative risk using the predicted 1-year mortality.

**Table 1:** The characteristics of the Index for Mortality Prediction After Cardiac Transplantation and the United Network for Organ Sharing risk scores and the risk stratification Scores

The IMPACT score		The UNOS score		The RSS	
Recipient variable	Points	Risk factors	Points	Risk factors	Points
Age >60 years	3	Recipient factors		Recipient age: >70 years	2.1
Serum bilirubin, mg/dl		Age >65 years	1	Recipient age: 55–70 years	1.2
0–0.99	0	Body mass index (kg/m^2^)		Previous cardiac surgery	1.3
1–1.99	1	30–35	1	Aetiology: congenital	2.3
2–3.99	3	>35	2	Aetiology: amyloidosis	1.8
≥4	4	MPAP >30 mmHg	1	Diabetes complicated by CVA	1.4
Creatinine clearance, ml/min		Total bilirubin		eGFR <33	2.8
≥50	0	1.5–1.9	1	eGFR 33–53	1.4
30–49	2	>1.9	2	Total bilirubin >2	1.7
<30	5	Creatinine		Intubated	1.8
Dialysis between listing and transplant	4	1.5–2.0	1	Hospitalized	1.2
Female sex	3	>2.0	2	RVAD-only	4.7
Heart failure aetiology		Previous transplant	2	ECMO	3.9
Idiopathic	0	Previous cancer	2	Extracorporeal LVAD	2.7
Ischaemic	2	Ventilator	2	Total artificial heart	2.4
Congenital	5	Mechanical circulatory support		Paracorporeal LVAD	2.7
Others	1	Noncontinuous-flow ventricular assist device	2	Hepatitis C (+) donor	2.1
Recent infection	3	Donor age: 50–55 years	1	Insulin-dependent donor	1.8
Intra-aortic balloon pump	3	Donor age: >55 years	2	Donor age: 50–59 years	1.7
Mechanical ventilation pretransplant	5	Ischaemia time >4 h	2	Donor age: 40–49 years	1.5
Race		Gender mismatch	1	Donor age: 30–39 years	1.3
White	0	Diabetes	1	Ischaemic time >6 h	1.7
African American	3	Total points	22	Ischaemic time 4–6 h	1.4
Hispanic	0			Female donor: male recipient	1.2
Others	0			Female donor: female recipient	1.2
Temporary circulatory support^a^	7			Total points	49.3
Ventricular assist device					
Older-generation pulsatile	3				
Newer-generation continuous^b^	5				
HeartMate II	0				
Total points	50				

a Temporary circulatory support includes extracorporeal membrane oxygenation and extracorporeal ventricular assist device support.

b Excluding HeartMate II.

CVA: cerebrovascular accident; ECMO: extracorporeal membrane oxygenation; eGFR: estimated glomerular filtration rate; IMPACT: Index for Mortality Prediction After Cardiac Transplantation; LVAD: left ventricular assist device; MPAP: mean pulmonary artery pressure; RSS: risk stratification score; RVAD: right ventricular assist device; UNOS: United Network for Organ Sharing.

The primary outcome was 1 year of observed all-cause mortality after a HTx; secondary outcomes included numbers of deaths (in-hospital and discharge to 1 year) after an HTx. We compared the observed and predicted mortality figures to assess the performances of the 3 risk scores. We assessed the model discrimination (statistical accuracy) by using the area under the receiver operating characteristics curve [18]. An area of 0.5 reflected no discrimination, and an area of 1.0 indicated a perfect predictor. Areas of >0.7 were generally thought to be useful. Additionally, binary logistic regression was used to evaluate associations between the risk scores and the postoperative 1-year mortality.

Patients were stratified into low-, medium- and high-risk groups based on IMPACT scores of <5, 5–10 and ≥10 points and UNOS scores of 0–2, 3–5 and >5, respectively, whereas the RSS stratified patients into low-, intermediate-, moderate-, elevated-risk and high-risk groups with risk scores of <2.55, 2.55–5.72, 5.73–8.13, 8.14–9.48 and >9.48, respectively. We calculated the observed mean 1-year mortality of different risk groups and compared those with the predicted 1-year mortality derived from the risk scoring models. To investigate the association between increasing scores and perioperative death, we also compared the in-hospital mortality of the different risk groups. Finally, Kaplan–Meier survival analysis was conducted with the 3 risk scores to assess the differences in survival between the different risk groups.

### Statistical analyses

Statistical analyses were performed with Statistical Product and Service Solutions version 21.0 (SPSS Inc., Chicago, IL, USA). Continuous variables were expressed as mean ± standard deviation and compared using the Student's *t*-test. Categorical variables were expressed as percentages and compared using the χ^2^ or the Fisher’s exact test. A conventional *P*-value of 0.05 or less was used to determine the level of statistical significance. All reported *P*-values were two-sided. Missing data were uncommon in our study, with a frequency of <1% missing for all variables. Kaplan–Meier curves were constructed to assess survival of the different risk groups. Survival curves were compared using the log-rank test.

## RESULTS

### Baseline characteristics of the study population

Over the study period, our centre had 451 patients who underwent a single-organ HTx. We excluded 21 patients younger than 18 years and 2 patients who lacked relative data; as a result, we included 428 patients. When we compared the demographic characteristics and risk factors between the Fuwai and the UNOS populations (Table 2), we found some significant differences. In the data provided by the IMPACT score, the Fuwai population was significantly younger than the UNOS population, with higher creatinine clearance and serum bilirubin levels, predominant idiopathic cardiomyopathy, less ischaemic heart disease for an HTx and a lower prevalence of hypertension. The Fuwai population were also more likely to have an intra-aortic balloon pump and extracorporeal membrane oxygenation as a bridge to a transplant; a left ventricular assist device was seldom used. Besides, Fuwai populations had longer ischaemic times on average. All differences were highly significant (*P *<* *0.01). In the data provided by the UNOS score, the Fuwai population had a lower prevalence for all recipient risk factors than the UNOS populations (*P *<* *0.05) mentioned in this score, whereas they had a higher prevalence of ischaemic time longer than 4 h than the UNOS population (*P *<* *0.01); the difference in the gender mismatch ratio between the 2 populations was not significant (*P *=* *0.53). Due to the unavailability of demographic characteristics in the RSS score, we only compared the risk strata condition between the 2 populations and found that the Fuwai population had more patients in the intermediate and moderate risk levels and fewer in the low-risk level compared to the UNOS population.

**Table 2:** Baseline characteristics of the study population

Variable^a^	FW hospital populations	UNOS populations (IMPACT score)	UNOS populations (UNOS score)	UNOS populations (RSS strata)	*P*-Value
Time	2015.01–2019.07	1997.01–2008.12	2005.01–2013.12	2001.01–2007.12	
Number	428	171 079	17 131	11 703	
Age, years	47.2 ± 12.6	52.1 ± 11.9	NA	NA	<0.01
Female sex	108/428 (25.2%)	4048/17 079 (23.7%)	NA	NA	0.46
Heart failure aetiology					
Idiopathic^b^	326/428 (76.2%)	7050/17 079 (42.1%)	NA	NA	<0.01
Ischaemic	65/428 (15.2%)	8118/17 079 (47.1%)	NA	NA	<0.01
Congenital	6/428 (1.4%)	400/17 079 (2.3%)	NA	NA	0.21
Others	31/428 (7.2%)	1511/17 079 (8.8%)	NA	NA	0.25
Hypertension	22/428 (5.1%)	5579/14 224 (39.2%)	NA	NA	<0.01
Diabetes mellitus	80/428 (18.7%)	3618/16 718 (21.6%)	NA	NA	0.21
Creatinine clearance,^c^ ml/min	79.3 ± 30.5	66.3 ± 25.8	NA	NA	<0.01
Serum bilirubin, mg/dl	1.38 ± 0.59	1.24 ± 2.11	NA	NA	<0.01
Preoperative mechanical ventilation	8/428 (1.9%)	456/17 079 (2.7%)	NA	NA	0.31
ECMO	12/428 (2.8%)	240/17 079 (1.4%)	NA	NA	0.02
Intra-aortic balloon pump	55/428 (12.9%)	927/17 079 (5.4%)	NA	NA	<0.01
Ventricular assist device					
Early generation^d^	0/428 (0.0%)	2,298/17 079 (13.5%)	NA	NA	<0.01
Late generation^e^	0/428 (0.0%)	374/17 079 (2.2%)	NA	NA	<0.01
Ischaemic time, h	4.84 ± 1.75	3.15 ± 1.04	NA	NA	<0.01
BMI >30 kg/m^2f^	2/62 (3.2%)	NA	2660/5784 (46.0%)	NA	<0.01
MPAP >30 mmHg^f^	32/62 (51.6%)	NA	3760/5784 (65.0%)	NA	0.03
Creatinine >1.5 mg/day^f^	29/62 (46.8%)	NA	3644/5784 (63.0%)	NA	<0.01
Ventricular assist device^f^^,g^	0/62 (0.0%)	NA	2603/5784 (45.0%)	NA	<0.01
Ischaemic time >4, h^h^	68/68 (100.0%)	NA	1168/1327 (88.0%)	NA	<0.01
Gender mismatch^h^	53/68 (77.9%)	NA	1075/1327 (81.0%)	NA	0.53
RSS risk group strata, *n* (%)					
Low risk	39 (9.1%)	NA	NA	3242 (27.7%)	<0.01
Intermediate risk	291 (68.0%)	NA	NA	6347 (54.2%)	<0.01
Moderate risk	84 (19.6%)	NA	NA	1543 (13.2%)	<0.01
Elevated risk	9 (2.1%)	NA	NA	310 (2.6%)	0.49
High risk	5 (1.2%)	NA	NA	261 (2.2%)	0.15

a Continuous data are presented as mean ± standard deviation and categorical data as number (%) of the overall cohort, unless otherwise specified.

b Idiopathic cardiomyopathy includes dilated cardiomyopathy, arrhythmogenic right ventricular cardiomyopathy, hypertrophic cardiomyopathy and restrictive cardiomyopathy.

c Based on a Cockcroft-Gault calculation: = {[140 − age (years)] × weight (kg)/[7.2 × plasma creatinine (mg/dl)]} × (0.85 if female).

d Early generation includes para- and intracorporeal pulsatile ventricular assist devices, including Abiomed AB5000, HeartMate I, XE and XVE, Thoratec IVAD (Thoratec Corp, Pleasanton, CA, USA); Toyobo (Toybo, Osaka, Japan); Novacor (World Heart Inc, Oakland, CA, USA); Medos (Medos, Stolberg, Germany); and LionHeart (Arrow International Inc, Reading, PA, USA).

e Later generation continuous ventricular assist devices including HeartMate II, Jarvik (Jarvik Heart Inc, New York, NY, USA); Micromed, Debakey (MicroMed Technology Inc, Houston, TX, USA); and VentrAssist (Ventracor, Sydney, Australia).

f Because the original publication on the United Network for Organ Sharing score provided only the data on major factors within the high-risk recipient group (total recipient score ≥3), we compared only the baseline characteristics in the high-risk recipient group between the 2 populations.

g The left VAD used in China include 2 domestic brands, including the EverHeart VAD and CH-VAD.

h The original publication on the United Network for Organ Sharing score provided only the data on major factors within the high-risk donor group (total recipient score ≥3), so we compared only the baseline characteristics in the high-risk donor group between the 2 populations.

BMI: body mass index; ECMO: extracorporeal membrane oxygenation; FW: Fuwai; IMPACT: Index for Mortality Prediction After Cardiac Transplantation; MPAP: mean pulmonary artery pressure; RSS: risk stratification score; UNOS: United Network for Organ Sharing; VAD: ventricular assist devices; NA: not available.

The donor information is shown in Table 3. All grafts in our study population were organs from brain-dead patients donated voluntarily. Compared to ISHLT populations, the donors in our population had longer ischaemia times, lighter weight, lower ratios of diabetes mellitus and a history of hypertension, a higher ratio of male donors and of male donors to female recipients and a lower ratio of female donors to male recipients. The distribution of the causes of death of the donors also differed between the 2 populations: The Fuwai population had a higher ratio of head trauma, stroke and central nervous system tumours, while having a lower ratio of anoxia and other causes. There was no significant difference between the 2 populations in terms of donor age and donor smoker history.

**Table 3:** Donor characteristics of the study population in the Fuwai Hospital compared with those of the study population of the International Society of Heart and Lung Transplantation registry

Donor characteristics^a^	FW hospital populations	ISHLT populations	*P*-Value
Time	January 2015–July 2019	January 2010–July 2018	
Number	428	36 883	
Age, years	36.0 (17.5–51.0)	35.0 (17.0–58.0)	0.19
Weight, kg	69.5 (50.0–85.0)	80.0 (57.0–115.2)	<0.01
Donor/recipient sex (% male)	92.8%/74.8%	67.9%/74.4%	<0.01
Female donor to male recipient	1.9%	16.0%	<0.01
Male donor to female recipient	19.9%	9.70%	<0.01
Diabetes mellitus	0.0%	3.5%	<0.01
History of smoking	13.8%	14.5%	0.67
Hypertension	11.2%	15.4%	0.02
Ischaemic time, h	4.4 (2.7–7.7)	3.2 (1.5–5.0)	<0.01
Donor cause of death			<0.01
Head trauma	58.9%	40.5%	
Stroke	23.6%	19.9%	
Anoxia	2.6%	21.5%	
Central nervous system tumour	3.0%	0.5%	
Others	11.9%	17.7%	

a Continuous data are presented as median (5th–95th percentiles) and categoric data are presented as percentage (%) of the overall cohort, unless otherwise specified.

FW: Fuwai; ISHLT: International Society for Heart and Lung Transplantation.

### Predictive accuracy of the Index for Mortality Prediction After Cardiac Transplantation, the United Network for Organ Sharing and the risk stratification scores

**Figure 1:**
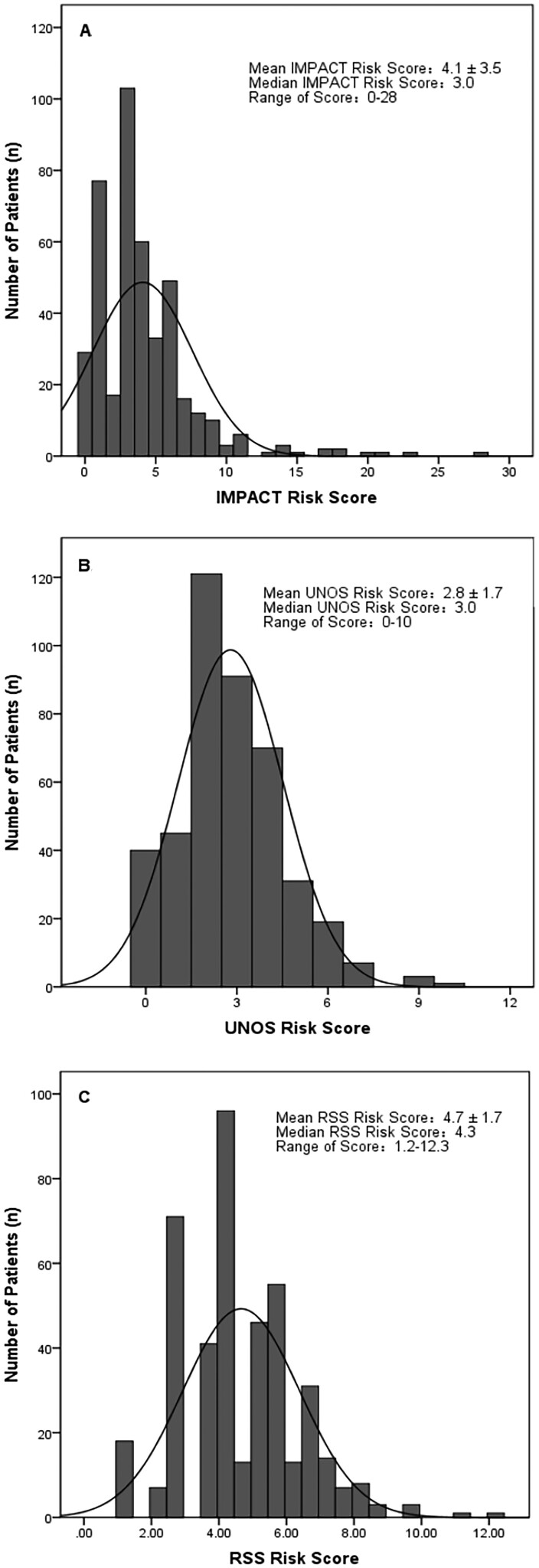
Distribution of risk scores based on the Index for Mortality Prediction After Cardiac Transplantation (**A**) score and the United Network for Organ Sharing (**B**) score and the risk stratification scores (**C**) in the study cohort.

The Fuwai cohort had a mean IMPACT score of 4.1 ± 3.5 (range: 0–28 points), a mean UNOS score of 2.8 ± 1.7 (range: 0–10 points) and a mean RSS of 4.7 ± 1.7 (range: 1.2–12.3 points). The distributions of the risk scores are shown in Fig. 1. In the logistic regression analysis, the IMPACT [odds ratio (OR), 1.25; 95% CI, 1.15–1.36; *P *<* *0.001] and the UNOS (OR, 1.68; 95% CI, 1.37–2.07; *P *<* *0.001) scores and the RSS (OR, 1.61; 95% CI, 1.30–2.00; *P *<* *0.001) were significant predictors of the 1-year mortality; the increasing risk scores of the 3 models were respectively associated with significant increases in the 1-year mortality risk.

**Figure 2:**
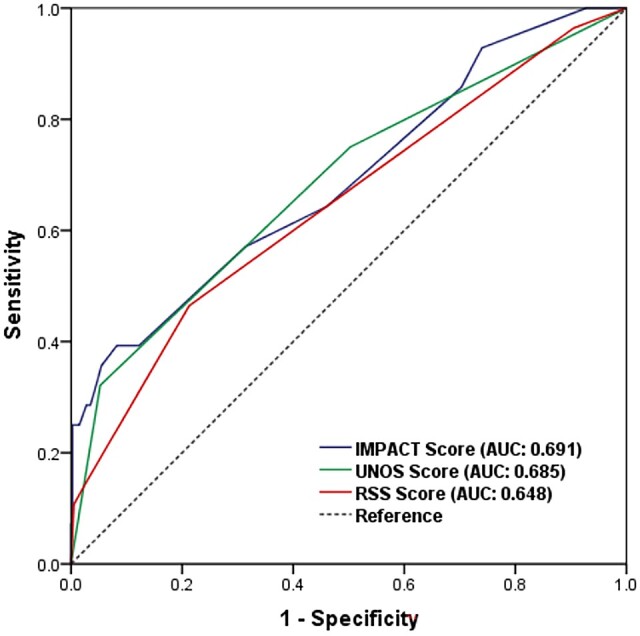
Receiver operator characteristics of the Index for Mortality Prediction After Cardiac Transplantation and the United Network for Organ Sharing Scores and Risk Stratification Scores for 1-year mortality (*n* = 428).

Among the 428 patients, there were 28 observed deaths, giving an overall observed mortality of 6.54%. We predicted a mortality of 10.59% (using the IMPACT score), a mortality rate of 10.74% (using the UNOS score) and a mortality rate of 12.89% (using the RSS), meaning that the IMPACT score had to be calibrated by a factor of 0.62, the UNOS score by a factor of 0.61 and the RSS by a factor of 0.51 to give an accurate representation of operative risk for the Fuwai population (Table 4). Figure 2 shows the predictive abilities of the 3 scores. The discriminatory ability of the IMPACT and UNOS scores and the RSS was acceptable but not satisfactory, with an area under the receiving curve of 0.691 with the IMPACT score, 0.685 with the UNOS score and 0.648 in the RSS for the cohort.

**Table 4:** Predictive ability of the index for mortality prediction based on the cardiac transplantation score

Type of score	Observed mortality (%)	Predicted mortality (%)	O:E	Area under the ROC curve
IMPACT score	6.54	10.59	0.62	0.691
UNOS score	6.54	10.74	0.61	0.685
RSS score	6.54	12.89	0.51	0.648

IMPACT: Index for Mortality Prediction After Cardiac Transplantation; O:E: ratio of observed to predicted mortality; ROC: receiver operating characteristic; RSS: risk stratification score; UNOS: United Network for Organ Sharing.

### Subanalysis of the post-heart transplant first-year mortality in different risk strata based on the Index for Mortality Prediction After Cardiac Transplantation and the United Network for Organ Sharing scores and the risk stratification scores

**Figure 3:**
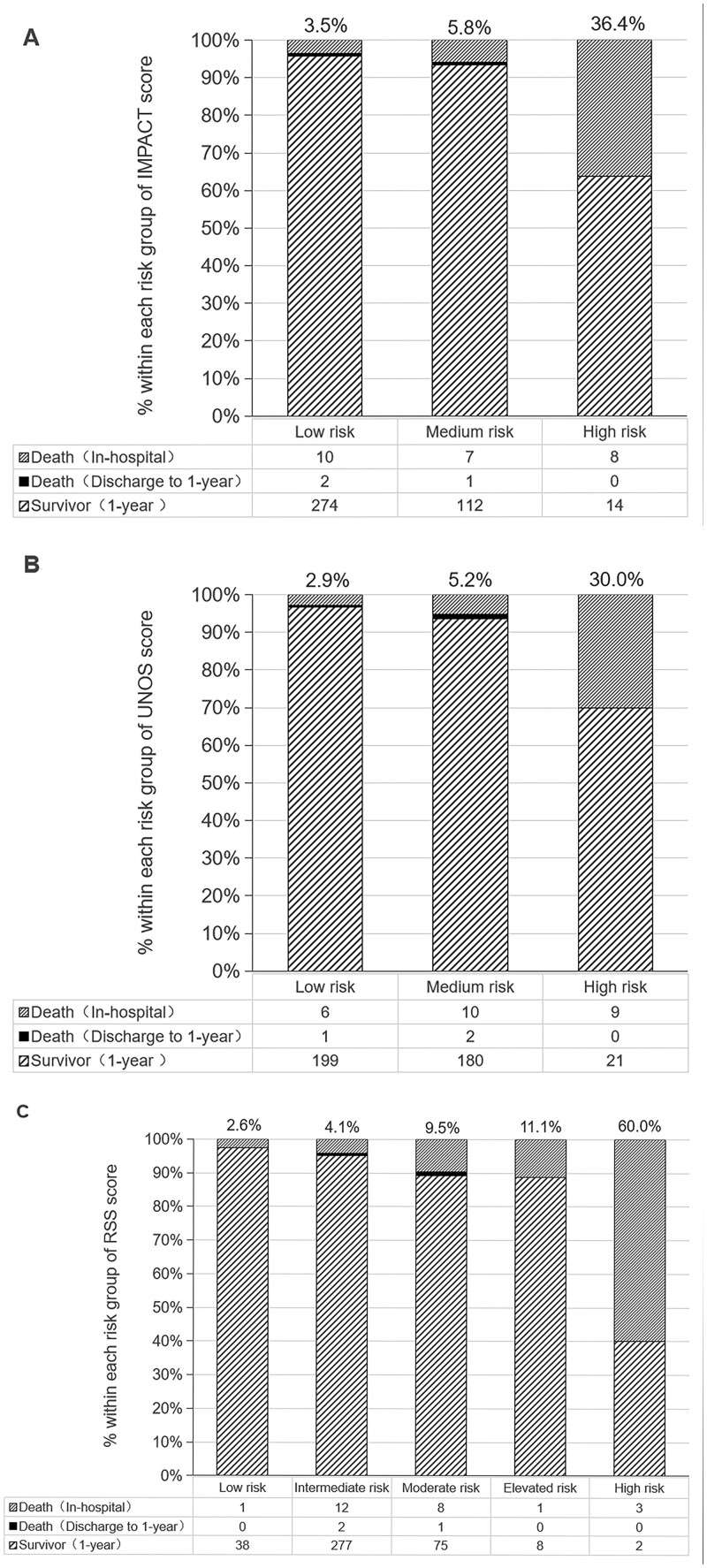
Number and proportion of deaths and survivors in the first year within different risk strata based on the Index for Mortality Prediction After Cardiac Transplantation (**A**), the United Network for Organ Sharing (**B**) risk scores and the risk stratification scores (**C**).

In the Fuwai cohort, the cumulative mortality of the first year after an HTx increased with increasing risk strata in the 3 risk scores, and most deaths during the first year were concentrated in the hospitalization period (Fig. 3). The causes of early death in Fuwai study population were provided in Appendix 1. There were only a few deaths among those patients who were still alive after discharge, and the difference between the risk strata was not significant (*P *>* *0.05). Table 5 shows the observed 1-year mortality and predicted 1-year mortality of different risk strata. Compared with the observed 1-year mortality, both the IMPACT and UNOS scores had higher predicted 1-year mortality in the low- and medium-risk groups while being lower in the high-risk group, and the RSS had higher predicted 1-year mortality in low-, intermediate-, moderate- and elevated-risk groups, while being lower in the high-risk group.

**Table 5:** Predicted and observed 1-year mortality within different risk strata based on the Index for Mortality Prediction After Cardiac Transplantation and the United Network for Organ Sharing risk scores and the risk stratification scores

Types of risk score	Survival by risk group	Predicted 1-year mortality (%)	Observed 1-year mortality (%)
IMPACT score	Low risk (score <5)	8.3	4.3
	Medium risk (score 5–10)	12.7	6.4
	High risk (score ≥10)	30.8	34.8
UNOS score	Low risk (score 0–2)	7.0	3.7
	Medium risk (score 3–5)	13.0	5.9
	High risk (score >5)	22.0	28.1
RSS score	Low risk (score <2.55)	6.2	2.6
	Intermediate risk (score 2.55–5.72)	10.8	4.8
	Moderate risk (score 5.73–8.13)	18.7	10.6
	Elevated risk (score 8.14–9.48)	33	11.1
	High risk (score >9.48)	53.0	60.0

IMPACT: Index for Mortality Prediction After Cardiac Transplantation; RSS: risk stratification score; UNOS: United Network for Organ Sharing.

### Kaplan–Meier analysis of the 1-year survival in different risk strata based on the Index for Mortality Prediction After Cardiac Transplantation, the United Network for Organ Sharing and the risk stratification scores

**Figure 4:**
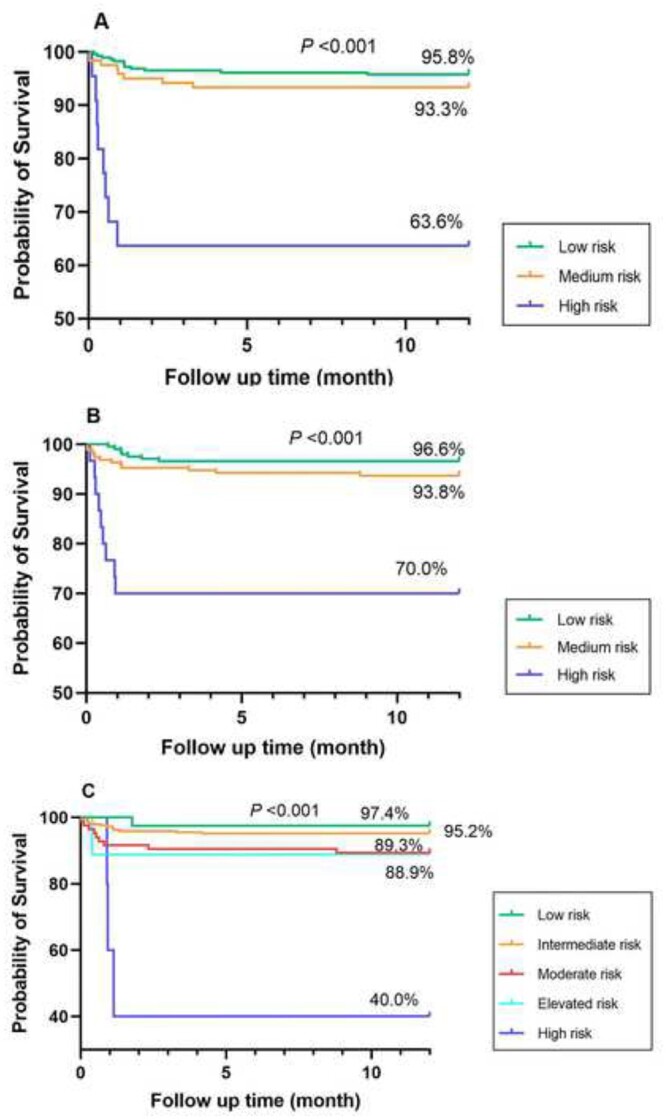
Kaplan–Meier 1-year survival curve for each risk group based on the Index for Mortality Prediction After Cardiac Transplantation scores (**A**), the United Network for Organ Sharing (**B**) risk scores and risk stratification scores (**C**).

The Kaplan–Meier analysis again showed lower risk strata correlated with improved survival. In the Kaplan–Meier analysis of the IMPACT score, patients in the low-risk group had a 32.2% lower 1-year cumulative survival than those in the high-risk group (95.8% vs 63.6%, respectively; *P *<* *0.001; Fig. 4A), whereas patients in the low-risk group had a higher but not a significant 1-year cumulative survival than those in the medium-risk group (95.8% vs 93.3%, respectively; *P *>* *0.05). The UNOS score had the same outcome (Fig. 4B). In the Kaplan–Meier analysis of the RSS, the 1-year cumulative survival decreased by the elevated risk strata and the low- (97.4%), intermediate- (95.2%), moderate- (89.3%) and elevated-risk groups (88.9%) all had a significantly higher 1-year cumulative survival than those in the high-risk group (40.0%), but the differences between the low and intermediate groups (97.4% vs 95.2%, respectively; *P *>* *0.05) and the moderate and elevated groups (89.3% vs 88.9%, respectively; *P *>* *0.05) were not significant.

## DISCUSSION

### Principal findings

In this study, we assessed the performance of the described IMPACT and UNOS risk scores and the RSS in predicting post-transplant 1-year outcomes within the Chinese population [10, 15, 17]. Through our verification, we found that these 3 risk scores were not particularly effective when applied to the Chinese population. We also showed that increased IMPACT and UNOS risk scores and the RSS were all predictors of 1-year mortality, but the discriminatory ability of the scores was not satisfactory; the area under the curve (AUC) of the 3 risk score models was smaller than 0.7. The 3 models overestimated overall mortality in the Fuwai population. Additionally, we found that the 3 scoring models could be used to identify high-risk patients before a transplant but could not be used to distinguish low- and medium-risk patients. In the prediction of 1-year mortality post-transplant, all risk score models overestimated the mortality in low- to medium-risk patients and underestimated mortality in high-risk patients. Thus, the 3 scores should be used with caution when predicting risk of HTx candidates in China.

### Performance of the Index for Mortality Prediction After Cardiac Transplantation and the United Network for Organ Sharing risk scores and the risk stratification scores

When we applied the 3 risk scoring models to other non-American populations, we came to similar conclusions. Weiss *et al.* [16] previously derived and validated the IMPACT score using UNOS data, and they also validated the IMPACT score using ISHLT registry data, which included 29 242 patients who received transplants between 2001 and 2010. They validated the fact that the IMPACT score could be used as a predictor of short- and long-term mortality after an HTx, but they did not show whether there was a difference in discrimination between the different cohorts. Nilsson *et al.* [13] used data from the ISHLT registry that included 68 085 patients who received transplants between 1994 and 2010 to validate the IMPACT scores and the RSS; they showed that the calibration for the RSS and IMPACT models was poor, both with an AUC of 0.61. Nguyen *et al.* [19] validated the IMPACT score in a French single-centre cohort, which included 414 patients; they found that the IMPACT risk score was not accurate for predicting post-HTx mortality (AUC = 0.58), whereas a subgroup analysis of patients undergoing an HTx after a VAD implant (*n* = 36) had a better performance, i.e. an IMPACT AUC of 0.72. Jasseron *et al.* [20] also validated the IMPACT score and used data from the French national transplant database CRISTAL that included 1776 patients; the calibration for the IMPACT score was also poor in their validation (AUC = 0.6). Sargut *et al.* [21] used data from 761 HTx recipients from the Euro-transplant region to validate the IMPACT score and the RSS; the AUC was 0.59 for IMPACT and 0.62 for RSS; they indicated that the IMPACT scores and the RSS were suitable for predicting post-transplant graft failure only in high- and low-risk cohorts. Besides, we did not find any studies that validated the UNOS score. Overall, existing studies showed that the IMPACT scores and the RSSs can be used as predictors of post-transplant mortality, but the calibration for the RSS and IMPACT models was unsatisfactory.

### Differences between the United Network for Sharing and the Fuwai populations in the use of the Index for Mortality Prediction After Cardiac Transplantation and the United Network for Organ Sharing and the risk stratification scores

The reasons why we were not able to accurately predict post-transplant outcomes in the Chinese population using the 3 risk scores were likely multifactorial. One possible reason was that the basic characteristics of the Chinese HTx population may be different from those of the UNOS population, which may have influenced the effectiveness of the 3 risk scores. From the recipients' side, the different and basic characteristics between 2 populations derived mainly from the difference in the HTx aetiology. Previous studies showed that the main reason for an HTx in the UNOS data was ischaemic cardiomyopathy, accounting for 47.1%, followed by idiopathic cardiomyopathy, accounting for 42.1% [17]. In China, the main cause was idiopathic cardiomyopathy, accounting for 76.2% in our centre, whereas ischaemic cardiomyopathy accounts for only 15.2%. Prior researchers mentioned that idiopathic cardiomyopathy usually occurs in adolescents and young adults [22], whereas ischaemic cardiomyopathy occurs mainly in older patients. Patients with ischaemic cardiomyopathy may have a poorer preoperative status because these patients usually have additional risk factors (e.g. advanced age, hypertension, obesity) and have a longer cardiovascular disease history than patients with idiopathic cardiomyopathy, which is associated with poorer peripheral vascular conditions and peripheral organ function. In summary, compared to Western populations, the Chinese populations were younger, with fewer risk factors and optimal, comprehensive organ function, which may be associated with a better prognosis for an HTx. From the donors' perspective, Chinese populations have a higher proportion of male donors and a lower proportion of donors with comorbidities (e.g. hypertension, diabetes), which may also be the reason for the better prognosis.

Another possible reason for the inaccuracy of the scores is that study populations received medical care at different time periods, and medical care significantly changed during these periods. The original data for the IMPACT scores were derived from patients who received transplants between 1997 and 2008 [17]; the RSS was derived from patients who received transplants between 2001 and 2007 [15], and the UNOS score was derived from patients who received transplants between 2005 and 2013 [10], whereas our study used data from patients who received transplants between 2015 and 2019. Due to recent advances in donor heart protection, surgery, anaesthesia, perfusion procedures, postoperative intensive care and immunosuppressive therapy [23–25], it is not surprising that the 3 risk-scoring models overpredicted overall mortality in contemporary Chinese HTx patients.

### Potential applications of Index for Mortality Prediction After Cardiac Transplantation and the United Network for Organ Sharing scores and risk stratification scores

Although the calibration for the 3 models was unsatisfactory, they can be used as a risk stratification tool to provide reference significance in the clinical decision-making process. The 3 risk scores in the cohort’s validation showed good stratification ability; relative risks of 1-year death clearly increased in the high-risk group. Therefore, all scoring models can be used to find patients at high risk and can be used as references when identifying the operative risk and targeting the treatment strategy during discussions of patients. Although high-risk patients may be unsuitable for an HTx, they can choose other therapeutic modalities to improve the possibility of survival and to obtain a higher quality of life, such as destination therapy, a left ventricular assist devices or palliative care [26]. Besides, the use of the risk scores may have a potential effect on the organ allocation process. According to the existing allocation policy, high-risk patients are more likely to be listed as urgent status and have priority in organ allocation. However, considering the high early mortality in high-risk HTx patients, the mortality of an HTx and the mortality of medication maintenance treatment should be weighed in the decision-making process for an HTx, to determine whether the priority assigned to high-risk patients is reasonable. In a word, the 3 risk scores can be used to classify the risk of Htx patients rather than to serve as an accurate predictor of 1-year mortality in individual patients.

### Other findings and future study

Another interesting finding was that most deaths during the first year were concentrated in the hospitalization period; among patients who successfully passed the perioperative period, who recovered and who were discharged from the hospital, the 1-year mortality was relatively low. This phenomenon was special in patients classified in the high-risk group, which reflected that high-risk recipients may have worse early outcomes compared to low-risk recipients. Besides, compared to Western populations, this phenomenon may also be related to the longer post-transplant hospital stays in our centre; thus, the patients in most cases were in a relatively stable condition before discharge. Therefore, early outcome indicators, i.e. perioperative mortality or in-hospital mortality, may be more representative of the impact of preoperative risk factors on transplant prognosis. Thus, we envision using in-hospital mortality as an outcome indicator for a risk score model, which may be more meaningful and valuable. Based on the preceding discussion and considering the on-going development of surgical and medical treatment, we suggest that it is necessary to construct a preoperative risk prediction model for a HTx that combines the characteristics of the Chinese population with perioperative death as the primary outcome and is suitable for Chinese and other Asian populations.

### Limitations

Our study has some limitations. First, our study was a single-centre retrospective observational study, limited by the inherent limitations of retrospective studies, such as accuracy and data integrity. Second, our study population was still relatively small compared to other studies that used a multicentre population to verify the risk models. We propose to validate the above 3 or more risk scores by using data from several Chinese large transplant centres in the future. Third, the prognosis of a HTx is largely influenced by donor factors; some donor factors, such as donor usage of vasoactive drugs, donor heart haemodynamic data and donor troponin values, should be included in the scoring system, so it is necessary to further explore the influence of other donor factors on risk assessment.

## CONCLUSION

This study validates the IMPACT and the UNOS scores and the RSS as predictors of 1-year mortality after an HTx. Unfortunately, all 3 risk scores had unsatisfactory discriminative powers that overestimated the observed mortality for the overall cohort. All 3 scoring models can be used to classify HTx-patient risks but not as accurate predictors of 1-year mortality in individual patients. Therefore, the 3 risk scoring tools should be used with caution in China. Creating a new model that can accurately predict outcomes in Chinese HTx patients is warranted.

## SUPPLEMENTARY MATERIAL

Supplementary material is available at *ICVTS* online.

## Supplementary Material

ivab380_Supplementary_Data

## ABBREVIATIONS

AUC: Area under the curve
HTx: Heart transplant
IMPACT: Index for Mortality Prediction After Cardiac Transplantation
ISHLT: International Society of Heart and Lung Transplantation
OR: Odds ratio
RSSs: Risk stratification scores
UNOS: United Network for Organ Sharing
