# Supplementary material for: Validation of existing risk scores for mortality prediction after a heart transplant in a Chinese population
Source: Interact Cardiovasc Thorac Surg. 2022 Jan 8;34(5):909–18. doi: 10.1093/icvts/ivab380 (PMC9070526; doi:10.1093/icvts/ivab380)
Supplement: ivab380_Supplementary_Data [file ivab380_supplementary_data.pdf]

Appendix 1. Cause of early death in Fuwai study population

Cause of early death (n=25)	Number (%)
Acute graft failure	8 (32.0%)
Multiple organ failure	4 (16.0%)
Infectious complications	5 (20.0%)
Acute rejection	4 (16.0%)
Acute digestive tract perforation	1(4.0%)
Acute gastrointestinal bleeding	1(4.0%)
Respiratory failure	1(4.0%)
Cerebral hemorrhages	1(4.0%)
